# Ventricular Tachycardia as a Presentation of Isolated Cardiac Sarcoidosis: How to Manage It When We Do Not See Granulomas

**DOI:** 10.7759/cureus.49163

**Published:** 2023-11-21

**Authors:** Luis René Puglla Sánchez, Begoña De Escalante Yangüela, Javier Escota-Villanueva, Juan Vallejo Grijalba, Darío J Samaniego Pesántez

**Affiliations:** 1 Cardiology, Hospital Clínico Universitario Lozano Blesa, Zaragoza, ESP; 2 Autoimmune Diseases Unit, Hospital Clínico Universitario Lozano Blesa, Zaragoza, ESP; 3 Internal Medicine, Hospital Clínico Universitario Lozano Blesa, Zaragoza, ESP; 4 Cardiology, Hospital Universitario Miguel Servet, Zaragoza, ESP

**Keywords:** positron emission tomography-computed tomography, cardiac sarcoidosis, ventricular tachycardia, magnetic resonance imaging, heart failure with reduced ejection fraction

## Abstract

A 47-year-old male was referred for rapid palpitations and an electrocardiogram compatible with sustained monomorphic ventricular tachycardia (VT) that required synchronized electrical cardioversion due to hemodynamic instability. After the initial clinical certainty, an etiological search is carried out.

The transthoracic echocardiogram (TTE) revealed moderate dilatation and left ventricular systolic dysfunction due to global hypokinesia. Coronary angiography did not show significant coronary stenosis. Cardiac magnetic resonance (CMR) guarantees a nonischemic dilated cardiomyopathy with moderate systolic dysfunction and a pattern of subepicardial and intramyocardial late gadolinium enhancement (LGE) in medial-lateral and median inferolateral segments. Lastly, a positron emission tomography-computed tomography (PET-CT) scan showed diffuse fixation of the radiotracer in the left ventricular (LV) walls, with greater uptake on the lateral and inferolateral surfaces of inflammatory origin. After ruling out other alternative pathologies and according to current diagnostic criteria, the clinical judgment of probable isolated cardiac sarcoidosis (ICS) is established.

An implantable cardioverter-defibrillator was implanted as secondary prevention of the acute arrhythmic event. Specific treatment for systolic dysfunction was prescribed, as well as immunosuppressive therapy with corticosteroids and methotrexate, after which the patient remained in clinical remission, with disappearance of active inflammation on cardiac imaging tests and progressive ventricular systolic function.

The initial diagnosis of isolated cardiac sarcoidosis can be complex and challenging, especially in those patients in whom the diagnosis of extracardiac sarcoidosis has not been previously established. The limitations of endomyocardial biopsy in this entity make it necessary to have a high index of clinical suspicion with the early use of new cardiac imaging techniques and to include this picture in the differential diagnosis of patients with sustained ventricular arrhythmias or left ventricular systolic dysfunction of nonspecific etiology clarified. Early initiation of aggressive immunosuppressive therapy has been shown to prevent disease progression and limit its potential cardiac complications.

## Introduction

Sarcoidosis is an autoimmune disease of unknown etiology with systemic involvement that is associated with the development of noncaseating granulomas. It can affect any organ, and cardiac involvement is estimated at 5%, although in autopsy series, it is confirmed in up to 25% of patients with systemic disease. Cardiac sarcoidosis (CS) can represent 13%-25% of the causes of death from this disease [1,2].

Isolated cardiac sarcoidosis (ICS) is a term coined for exclusive cardiac involvement, with high mortality if it is not suspected or diagnosed on time, and it is a frequent cause of sudden cardiac death in autopsy studies. Myocardial inflammation, granuloma formation, and scarring changes can cause conduction disturbances, especially auricular-ventricular atrioventricular blocks, sustained ventricular arrhythmias, ventricular systolic dysfunction, and heart failure in these patients [3,4].

The current gold standard to confirm cardiac involvement by sarcoidosis is based on the histopathological finding of confirmed noncaseating granulomas in an endomyocardial biopsy or to recognize cardiac symptoms or signs in patients with sarcoidosis histologically confirmed in other organs [4,5]. Establishing the diagnosis of ICS without histopathological confirmation, based on the combination of clinical data, cardiac imaging techniques, and favorable response to its specific treatment, represents a paradigm shift in the current management of this entity and a reasonable alternative in specific cases.

## Case presentation

**Figure 1:**
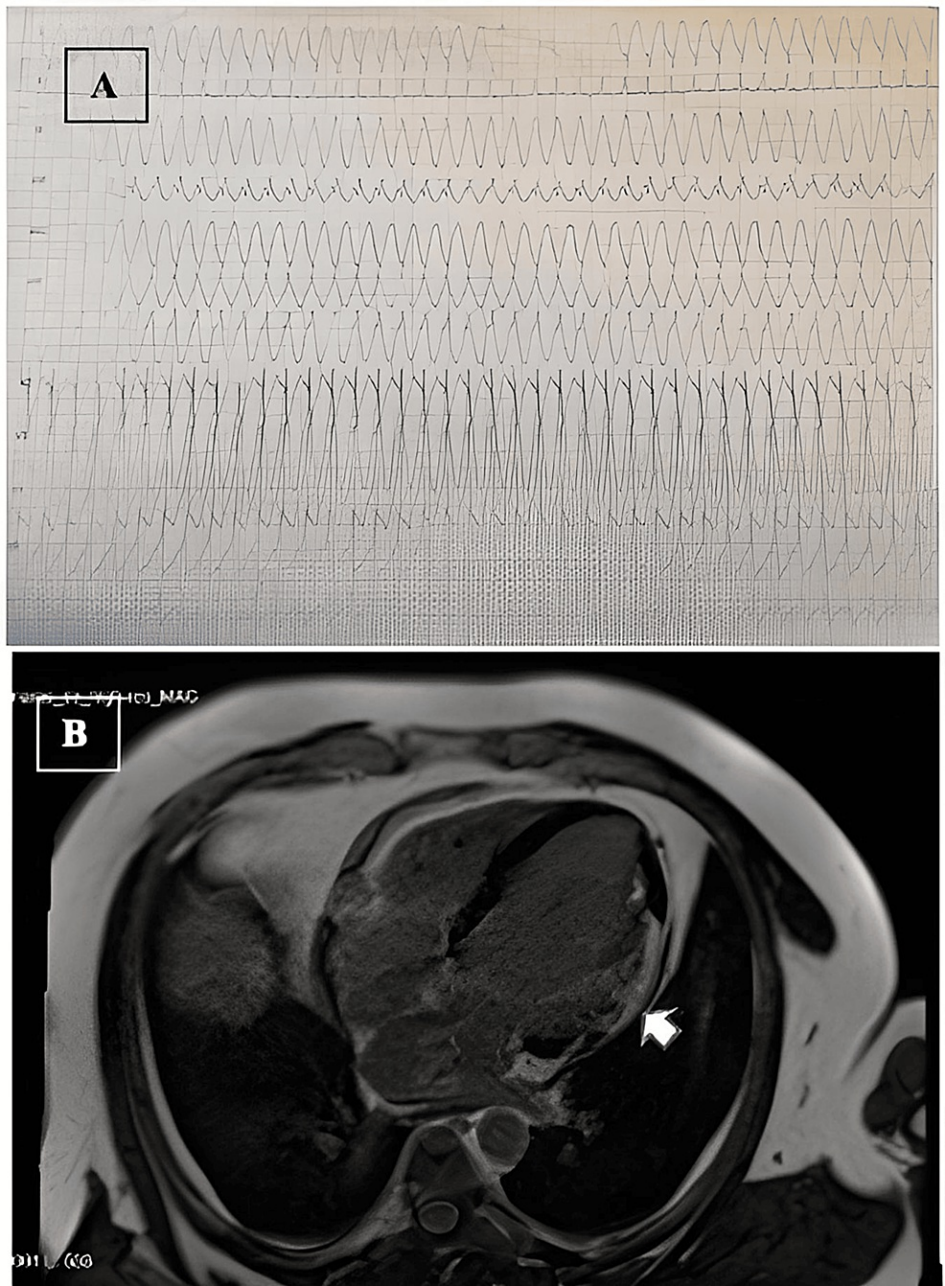
A: SMVT with right bundle branch block morphology in a patient with ICS. B: CMR with subepicardial LGE in the midposterior segment (white arrow). SMVT: sustained monomorphic ventricular tachycardia, ICS: isolated cardiac sarcoidosis, CMR: cardiac magnetic resonance, LGE: late gadolinium enhancement

A 47-year-old male, with no toxic habits and no relevant medical or family history, was referred to the emergency department due to palpitations and thoracic discomfort while working in the office. An electrocardiogram (Figure 1A) was performed, documenting sustained monomorphic ventricular tachycardia (VT) with right bundle branch block and inferior axis morphology. Due to hemodynamic instability, we proceeded to perform synchronized electrical cardioversion at 200 J biphasic, passing to sinus rhythm with repolarization abnormalities, presenting T wave inversion in inferior and high lateral leads.

After initial clinical stabilization, different cardiology tests were performed to find arrhythmic substrates. Both physical examination and blood tests did not show relevant findings (including the determination of angiotensin-converting enzyme). A transthoracic echocardiogram (TTE) showed moderate dilatation and left ventricular systolic dysfunction (left ventricular ejection fraction (LVEF): 37%) due to global hypokinesia, without myocardial scarring. Coronary angiography revealed the absence of significant coronary lesions, which ruled out a coronary ischemic cause.

Subsequently, a cardiac magnetic resonance (CMR) was performed, which confirmed moderate systolic dysfunction (LVEF: 40%) with a pattern of late gadolinium enhancement (LGE) predominantly subepicardial and intramyocardial in the anterolateral and inferolateral medial segments of LV (Figure 1B), suggestive of focal fibrosis in these locations. An ischemic etiology was definitively excluded, and we ruled out other diseases such as hemochromatosis, Fabry disease, autoimmune diseases with negative autoantibodies, infectious diseases with negative serologies, and Chagas disease. There was no recent history of infections.

**Figure 2:**
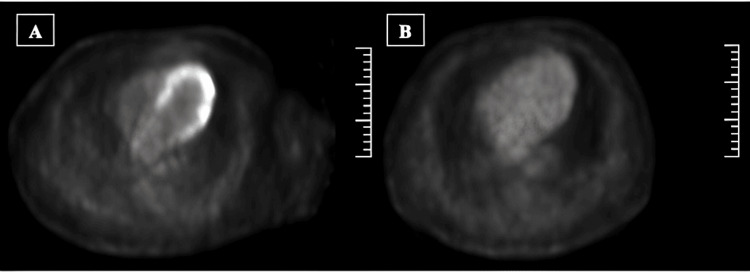
A: 18F-PET-CT with radiotracer uptake in LV walls in the lateral and posterior region. B: Control 18F-PET-CT with no uptake of the radiotracer after two years of starting treatment. 18F-PET-CT: fluor-18 positron emission tomography-computed tomography, LV: left ventricle

Because of the clinical presentation (sustained ventricular arrhythmia) and exclusion of other diseases such as infectious myocarditis, we consider that cardiac sarcoidosis was the most plausible diagnosis. For this reason, a fluor-18 positron emission tomography-computed tomography (18F-PET-CT) was performed to establish the presence of an active myocardial inflammatory phase and try to show evidence of extracardiac involvement (potentially for biopsy). PET showed diffuse fixation of the radiotracer in the walls of the left ventricle, with greater uptake on the lateral and inferolateral sides in relation to inflammatory activity in this area (Figure 2A). Likewise, the presence of extracardiac hypermetabolism was ruled out.

In a patient with nonischemic dilated cardiomyopathy with moderate systolic dysfunction that debuted as sustained monomorphic VT, with a predominantly anterolateral and inferolateral subepicardial LGE pattern on CMR and intense myocardial uptake on 18F-PET-CT in those segments without extracardiac involvement, the diagnosis of a probable ICS in the phase of active inflammation was considered. For definitive confirmation, an endomyocardial biopsy was ordered, but the patient refused to proceed, with no alternative biopsy in another organ in view of the PET-CT result.

The placement of an implantable cardioverter defibrillator (ICD) was discussed. Finally, it was decided to implant ICD as secondary prevention related to sustained VT with hemodynamic instability and the existence of intense myocardial fibrosis in the ventricular wall.

Regarding pharmacological treatment, in agreement with the Autoimmune Diseases Unit, we started immunosuppressive therapy with prednisone 30 mg daily and methotrexate 15 mg weekly. In addition, the patient received neurohormonal treatment for left ventricular systolic dysfunction (sacubitril/valsartan, eplerenone, bisoprolol, and empagliflozin) titrated to their effective doses. In the clinical follow-up at two years, the patient has remained asymptomatic with no cardiovascular events, without ICD therapies needed, and with progressive improvement in systolic function, reaching an LVEF of 55% in the last transthoracic echocardiography control. Likewise, in the repeated PET-CT controls, a progressive decrease in myocardial uptake was observed until its complete remission after two years of treatment (Figure 2B), allowing de-escalation of the corticoids and withdrawal of methotrexate after five years of treatment.

## Discussion

Isolated cardiac sarcoidosis (ICS) is defined by histological changes due to noncaseating granulomas with exclusive involvement of the heart, in the absence of extracardiac sarcoid disease [6,7]. Its diagnosis is complex, since the endomyocardial biopsy, although highly specific, is only positive in 25% of cases due to nonhomogeneous involvement, but patchy in most cases [8,9].

In ICS, the presence of ventricular arrhythmias seems to be due to scarring from the inflammatory damage of sarcoid granulomas and is associated with high morbidity and mortality. For this reason, it is important to include ICS in the differential diagnosis of patients with “de novo” monomorphic VT in whom coronary ischemic etiology has been ruled out, even without a known history of sarcoidosis, like our case [10].

Although endomyocardial biopsy is the gold standard for the diagnosis of this entity, its performance has several risks, and its utility is limited by its low sensitivity. In this context, the appearance of advanced cardiac imaging tests such as PET-CT and CMR represents an indisputable advance in the noninvasive diagnosis of this disease [11] to confirm the diagnosis of CS.

The Japanese Society of Cardiology already includes, among its major diagnostic criteria for CS, the typical uptake pattern of this entity in the most advanced cardiac imaging tests such as 18F-PET-CT or CMR. Other major diagnostic criteria of this society that are also present in our case were as follows: the presence of sustained ventricular arrhythmias with no other alternative cause and left ventricular systolic dysfunction of unknown etiology [6]. The Heart Rhythm Society has added a favorable response to steroid therapy as an additional diagnostic criterion, which should also be considered in suspected CS in the absence of a confirmatory biopsy [8,10]. However, it is evident that both scientific societies still consider biopsy as an element essential in the definitive diagnosis of CS.

As we have already mentioned, it is important to carry out an exhaustive differential diagnosis with other diseases that present with ventricular arrhythmias and abnormal uptake in imaging tests. Pathologies such as infectious myocarditis, arrhythmogenic right ventricular cardiomyopathy, and other infiltrative pathologies such as Fabry disease, hemochromatosis, or infections such as Chagas disease, HIV, and other autoimmune diseases must be ruled out, as was done in our case [4].

In the management of this condition, the treatment of active myocardial inflammation is essential, for which there are studies that support the initiation of early corticosteroid therapy and immunosuppressive therapy with methotrexate, thereby reducing the inflammatory response, stopping the appearance of myocardial fibrosis, and stopping the progression of the disease. Subsequent monitoring of response to treatment should include cardiac imaging tests such as 18F-PET-CT, which is especially sensitive for establishing the degree of myocardial inflammation and the presence of sarcoidosis in the active phase [11].

On the other hand, the management of the evolutionary complications of the disease, such as left ventricular systolic dysfunction and sustained ventricular arrhythmias, must not be forgotten. In our case, neurohormonal treatment has been maintained with the four current pharmacological pillars that include the use of sacubitril/valsartan, beta-blocker, sodium-glucose cotransporter 2 (SGLT2) inhibitors, and mineralocorticoid receptor antagonist. Although treatment for ICS has not been evaluated in randomized studies, it is assumed that early immunosuppressive treatment and treatment of derived cardiac complications (left ventricular systolic dysfunction and ventricular arrhythmias) could clearly reduce symptoms and improve short- and long-term prognosis [12,13].

## Conclusions

The initial clinical presentation of a patient with ICS can be challenging, especially if there is no known prior diagnosis of extracardiac sarcoidosis. It is important to take this pathology into account in our clinical practice in order to be able to apply, through multidisciplinary collaboration, correct guidelines for action that do not delay diagnosis or treatment in these patients and thus be able to improve their quality of life and life expectancy.
